# *In Vitro* Antimicrobial and Modulatory Activity of the Natural Products Silymarin and Silibinin

**DOI:** 10.1155/2015/292797

**Published:** 2015-03-11

**Authors:** Dayanne Rakelly de Oliveira, Saulo Relison Tintino, Maria Flaviana Bezerra Morais Braga, Aline Augusti Boligon, Margareth Linde Athayde, Henrique Douglas Melo Coutinho, Irwin Rose Alencar de Menezes, Roselei Fachinetto

**Affiliations:** ^1^Programa de Pós-Graduação em Ciências Biológicas, Bioquímica Toxicológica, Universidade Federal de Santa Maria, 97105-700 Santa Maria, RS, Brazil; ^2^Laboratório de Microbiologia e Biologia Molecular, Universidade Regional do Cariri (URCA), 63100-000 Crato, CE, Brazil; ^3^Departamento de Farmácia Industrial, Universidade Federal de Santa Maria, 97105-700 Santa Maria, RS, Brazil; ^4^Laboratório de Microbiologia e Biologia Molecular, Departamento de Química Biológica, Universidade Regional do Cariri, 63100-000 Crato,CE, Brazil; ^5^Programa de Pós-Graduaçãoo em Farmacologia, Universidade Federal de Santa Maria, 97105-700 Santa Maria, RS, Brazil

## Abstract

Silymarin is a standardized extract from the dried seeds of the milk thistle (*Silybum marianum* L. Gaertn.) clinically used as an antihepatotoxic agent. The aim of this study was to investigate the antibacterial and antifungal activity of silymarin and its major constituent (silibinin) against different microbial strains and their modulatory effect on drugs utilized in clinical practice. Silymarin demonstrated antimicrobial activity of little significance against the bacterial strains tested, with MIC (minimum inhibitory concentration) values of 512 *µ*g/mL. Meanwhile, silibinin showed significant activity against *Escherichia coli* with a MIC of 64 *µ*g/mL. The results for the antifungal activity of silymarin and silibinin demonstrated a MIC of 1024 *µ*g/mL for all strains. Silymarin and silibinin appear to have promising potential, showing synergistic properties when combined with antibacterial drugs, which should prompt further studies along this line.

## 1. Introduction

Microbial infections have become one of the principal problems of public health in the world, affecting all countries, developing or developed. It can be related to the process of natural selection in bacterial development or the natural consequence of the adaptation of bacteria to exposure to antibiotics in the course of the indiscriminate use of antibiotics in humans and animals. Various cases related to resistance have been reported, including methicillin-resistant* Staphylococcus aureus* (MRSA) [1], penicillin-nonsusceptible* Streptococcus pneumoniae* (PNSSP), vancomycin-resistant enterococci (VRE), extended spectrum beta-lactamase- (ESBL-) producing Enterobacteriaceae, and* Candida sp* resistant to imidazoles.

*Staphylococcus aureus* is a common cause of cutaneous and soft tissue infections, as well as invasive illness, such as bacteremia, septic arthritis, osteomyelitis, and necrotizing pneumonia [2, 3].* Escherichia coli *is a Gram-negative bacillus that causes infections, especially neonatal, such as meningitis and septicemia, and even diarrheal diseases, in the whole world, particularly affecting children up to 5 years old.* E. coli *is typical of the intestinal flora and commensal of the vaginal flora [4, 5]. Also,* Pseudomonas aeruginosa* has often been associated with occurrence of hospital infections and antibiotic resistance events [6, 7].

Microbial resistance is thus problematic for public health, especially coupled to virulence potential of these multiresistant pathogens. Accordingly, we should emphasize the relevance of the discovery of new drugs with antimicrobial capacity and/or showing synergism with drugs already employed in clinical practice [8]. Thus, in the last years, there has been increased use of plants and their derivatives as an alternative modality in the treatment of various diseases, including infections caused by microorganisms [9].

Infections by yeasts of* Candida* occur on cutaneous and mucosal surfaces, but, in some cases, they become severe by causing systemic infection, especially in immunocompromised patients. Moreover, therapeutic options are still limited, particularly in treating resistant pathogens [10]. Additionally, the indiscriminate use of broad spectrum antibiotics has contributed to the development of fungal infections [11].

Silymarin is a standardized extract from the dried seeds of the milk thistle (*Silybum marianum *L. Gaertn.), family Asteraceae [12]. Silymarin contains approximately 70–80% flavonolignans and 20–30% nonidentified oxidized polyphenolic compounds fraction. The mixture of flavonolignans consists mainly of silybin (silibinin), the major bioactive component of the extract, and isomeric isosilybin, silychristin, and silydianin and two flavonoids (taxifolin and quercetin) [13, 14]. Silibinin currently is recommended for use in alcoholic liver disease. Ethanol induces free radical formation through multiple pathways, resulting in steatohepatitis and cirrhosis with chronic use [15, 16].

Silymarin has clinical use as antihepatotoxic agent [17], has anti-inflammatory properties [18], and is antitumor [19], antifibrotic, and cytoprotective [20]. Studies have reported the synergistic activity of silibinin when combined with ampicillin and gentamicin against bacteria that attack the oral cavity [21]. However, there are few works that have evaluated the antimicrobial capacity of silymarin and silibinin, demonstrating the need to extend the study of their therapeutic use in this regard.

The objective of this work was to investigate silymarin and its major component, silibinin, for possible antimicrobial effects and drug-modifying activity when combined with antibacterial and antifungal drugs commonly used in the clinic and also to compare the activity of the two agents.

## 2. Materials and Methods

### 2.1. Quantification of Compounds by HPLC-DAD

Reverse phase chromatographic analyses were carried out under gradient conditions using C_18_ column (4.6 mm × 250 mm) packed with 5 *μ*m diameter particles. The mobile phase was water containing 1% formic acid (A) and methanol (B), and the composition gradient was 15% of B for 10 min and was changed to obtain 20%, 30%, 50%, 60%, 70%, 20%, and 10% B at 20, 30, 40, 50, 60, 70, and 80 min, respectively, following the method described by Boligon et al. [22] with slight modifications. Silymarin extract was analyzed at a concentration of 2.4 mg/mL; the identification of silybin (A and B), gallic acid, and caffeic acid was performed by comparing their retention time and UV absorption spectrum with those of the commercial standards. The flow rate was 0.6 mL/min, the injection volume was 40 *μ*L, and the wavelength was 254 nm for gallic acid, 280 nm for silybin (A and B), and 327 nm for caffeic acid. All the samples and mobile phase were filtered through 0.45 *μ*m membrane filter (Millipore) and then degassed by ultrasonic bath prior to use. A stock solution of standards references was prepared in the HPLC mobile phase at a concentration range of 0.030–0.250 mg/mL. The chromatography peaks were confirmed by comparing DAD (Diode Array Detector) retention time with those of reference standard and by DAD spectra (200 to 500 nm). Calibration curve for gallic acid is *Y* = 11945 + 1268.4 (*r* = 0.9997), for caffeic acid is *Y* = 13407 + 1361.8 (*r* = 0.9992), for silybin A is *Y* = 12683 + 1185.9 (*r* = 0.9999), and for silybin B is *Y* = 13045x + 1376.1 (*r* = 0.9995). All chromatography operations were carried out at ambient temperature and in triplicate. The limit of detection (LOD) and limit of quantification (LOQ) were calculated based on the standard deviation of the responses and the slope using three independent analytical curves. LOD and LOQ were calculated as 3.3 and 10 *σ*/*S*, respectively, where *σ* is the standard deviation of the response and *S* is the slope of the calibration curve.

### 2.2. Preparation of Stock Solution and Test Solutions

Stock solutions of silymarin and silibinin were prepared at a concentration of 10 mg/mL in 1 mL of dimethylsulfoxide (DMSO). Using this concentration, the compounds were diluted in 1 mL of sterile distilled water to obtain a concentration of 1024 *μ*g/mL (test solution).

### 2.3. Fungal and Bacterial Strains

The minimal inhibitory concentration (MIC) of silymarin and silibinin was determined using the bacterial strains* Escherichia coli *25922,* Staphylococcus aureus *25923, and* Pseudomonas aeruginosa *9027 and the fungal strains* Candida albicans* 62,* C. krusei *02, and* C. tropicalis *20. All strains were obtained from the Clinical Mycology Laboratory of the Federal University of Paraiba. In the antibiotic-modifying assays, we used the multiresistant bacterial strains from the clinical isolates* Pseudomonas aeruginosa* 03,* Escherichia coli *06, and* Staphylococcus aureus *10 and the standard yeasts* Candida albicans*, INCQS 40006,* C. krusei*, INCQS 40095, and* C. tropicalis*, INCQS 400042.

We utilized the following culture media for bacteria: heart infusion agar (HIA; Difco Laboratories Ltda.) and brain heart infusion broth (BHI at 10% as indicated by the manufacturer; Acumedia Manufacturers Inc.). Sabouraud dextrose broth was used for fungi. All culture media were prepared following the manufacturer's instructions. Fungal and bacterial cultures were maintained at 4°C in HIA. Before the tests, the strains were passaged using the above media and incubated at 37°C for 24 h. The plated strains were inoculated into BHI broth and again incubated at 37°C for 24 h. A small aliquot of the cultivated inoculum was removed and diluted in sterile saline to give turbidity equivalent to 0.5 on the McFarland scale, corresponding to 10^5^ CFU/mL [23]. The resistance profile and origin of the bacterial strains are described in Table 1.

### 2.4. Drugs

The antibacterial drugs utilized were amikacin, gentamicin, ciprofloxacin, and imipenem/cilastatin sodium, and the antifungals employed were mebendazole and nystatin (Sigma Co., St. Louis, USA), at an initial concentration of 2500 *μ*g/mL and 1024 *μ*g/mL, respectively. All drugs were dissolved in sterile water. Silymarin powder was obtained commercially from DEG importation (Santa Maria, Brazil). Silibinin was obtained from Sigma Co. The reagent sodium resazurin was utilized as the indicator of bacterial growth; it was also obtained from Sigma Co. and stored at 4°C away from light. In reading the assay, a color change from blue to pink due to the reduction of resazurin indicated bacterial growth [24].

### 2.5. Determination of Minimal Inhibitory Concentration (MIC)

Antimicrobial activity of the assayed products was determined by the microdilution assay. A volume of 100 *μ*L of 10% BHI medium was added to each well of a 96-well microplate and 100 *μ*L of the test product was used to do a twofold serial dilution giving concentrations of 512 to 8 *μ*g/mL. Next, 100 *μ*L of the bacterial or yeast suspension was added to all wells except the negative control or blank. The negative control contained 100 *μ*L of 10% BHI medium and 100 *μ*L of test product. Meanwhile, the positive control contained the bacterial or yeast suspension and 10% BHI. The plates were placed in an incubator for 24 h at 37°C [25]. Bacterial growth was determined utilizing resazurin, while fungal growth was evaluated according to turbidity. The assays were done in triplicate. MIC was defined as the lowest concentration at which no growth was observed in accordance with NCCLS [23].

### 2.6. Test for Antibiotic-Modifying Activity

Silymarin and silibinin were tested for possible antibiotic-modifying activity by their combination with the antibacterial and antifungal drugs listed above, according to the method proposed by Coutinho et al. [26], where the test products were used at a subinhibitory concentration (MIC/8).

### 2.7. Statistical Analysis

Each experiment was performed six times and the results were normalized by calculation of geometric mean values. Error deviation and standard deviation of the geometric mean were revealed. Statistical analyses were performed using GraphPad Prism, version 5.02. Differences between treatment with antibiotics in the absence and in the presence of the products were examined using two-way analysis of variance (ANOVA). The differences mentioned above were analyzed by Bonferroni posttest and they were considered statistically significant when *P* < 0.05.

## 3. Results

### 3.1. HPLC Analysis

**Figure 1:**
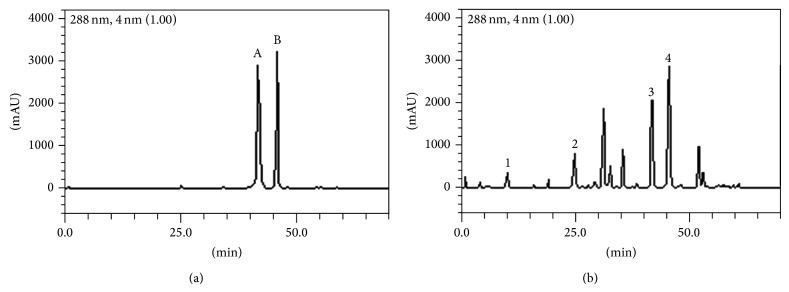
Representative high performance liquid chromatography profile of Silymarin, detection UV was at 288 nm. Gallic acid (peak 1), caffeic acid (peak 2), silybin A (peak 3), and silybin B (peak 4) ((a) and (b)).

HPLC fingerprinting of silymarin extract revealed the presence of gallic acid (*t*_*R*_ = 10.19 min; peak 1), caffeic acid (*t*_*R*_ = 24.97 min; peak 2), silybin A (*t*_*R*_ = 42.17 min; peak 3), and silybin B (*t*_*R*_ = 45.89 min; peak 4). Calibration curve for gallic acid is *Y* = 11945 + 1268.4 (*r* = 0.9997), for caffeic acid is *Y* = 13407 + 1361.8 (*r* = 0.9992), for silybin A is *Y* = 12683 + 1185.9 (*r* = 0.9999), and for silybin B is *Y* = 13045x + 1376.1 (*r* = 0.9995) (Table 2; Figure 1).

### 3.2. Antibacterial Activity and Modulation of Antibiotic Activity by Silymarin Extract and Silibinin

**Figure 2:**
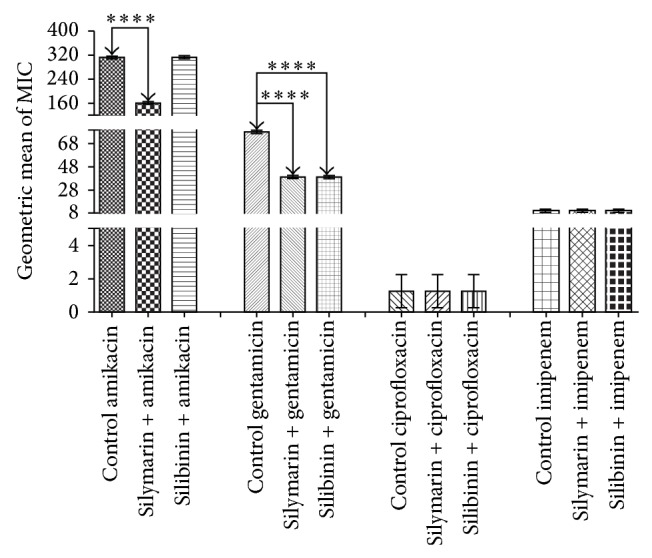
MIC (*μ*g/mL) of the antibiotics in the absence and presence of silymarin and silibinin at subinhibitory concentrations for* E. coli* strain EC06.

**Figure 3 fig3:**
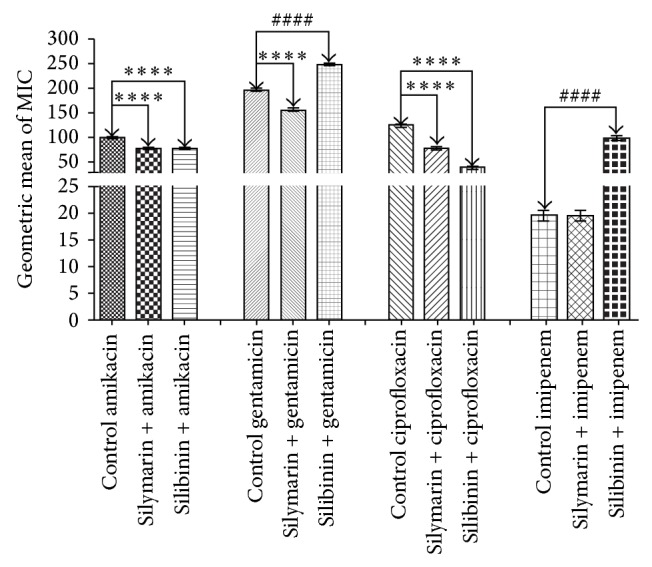
MIC (*μ*g/mL) of the antibiotics in the absence and presence of silymarin and silibinin at subinhibitory concentrations for* P. aeruginosa* strain PA03.

**Figure 4:**
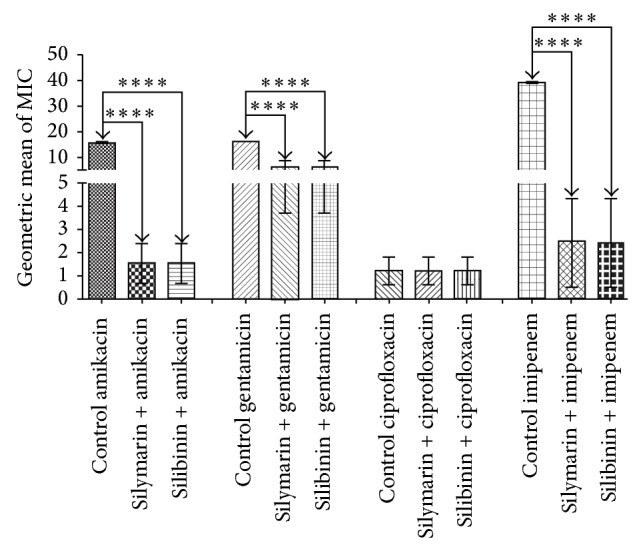
MIC (*μ*g/mL) of the antibiotics in the absence and presence of silymarin and silibinin at subinhibitory concentrations for* S. aureus *strain SA10.

Silymarin demonstrated antimicrobial activity that was clinically irrelevant, with MIC values of 512 *μ*g/mL. The results demonstrating the modulatory antibiotic activity of silymarin and silibinin are demonstrated in Figures 2 [Fig fig3]–4 and the silymarin at a concentration of 64 *μ*g/mL was combined with the antibiotics. Silibinin showed a MIC of 1024 *μ*g/mL and was thus clinically irrelevant for the strains* Staphylococcus aureus* ATCC 25923 and* Pseudomonas aeruginosa *ATCC 9027, so a concentration of 128 *μ*g/mL was used in drug-modifying assays. However, for* Escherichia coli *ATCC 25922, the MIC was 64 *μ*g/mL, and thus, a concentration of 8 *μ*g/mL was used in drug-modifying assays.

Silymarin and silibinin demonstrated antifungal activity with MIC value of 1024 *μ*g/mL for all strains, and thus, a concentration of 128 *μ*g/mL was used for both products to evaluate antibiotic-modifying activity.

Silymarin demonstrated significant synergistic activity in modulating the effect of aminoglycosides against* E. coli *(*P* < 0.001), reducing the MIC from 312.5 to 156.25 *μ*g/mL for amikacin and from 78.125 to 39.06 *μ*g/mL for gentamicin. Silibinin showed similar synergism when combined with gentamicin (*P* < 0.001), lowering the MIC from 78.125 to 39.06 *μ*g/mL when compared to the control. Silymarin and silibinin showed significant synergism in the presence of the antibiotics amikacin with a reduction in MIC from 78.125 to 39.06 *μ*g/mL (*P* < 0.001) and ciprofloxacin with a reduction in MIC from 78.125 to 39.06 *μ*g/mL (*P* < 0.001) against* P. aeruginosa *compared to the control. Silymarin also demonstrated a significant synergistic effect when combined with gentamicin lowering the MIC from 156.25 to 78.125 *μ*g/mL (*P* < 0.001) in relation to the control.

It is important to mention that silibinin showed an antagonistic effect when combined with gentamicin and imipenem. But against* S. aureus*, silymarin and silibinin displayed substantial synergistic activity when combined with the antibiotics amikacin, reducing the MIC from 19.53 to 1.22 *μ*g/mL (*P* < 0.001), gentamicin, lowering the MIC from 19.53 to 9.76 *μ*g/mL (*P* < 0.001), and imipenem, reducing the MIC from 39.06 to 2.44 *μ*g/mL (*P* < 0.001), compared to the control.

**Figure 5:**
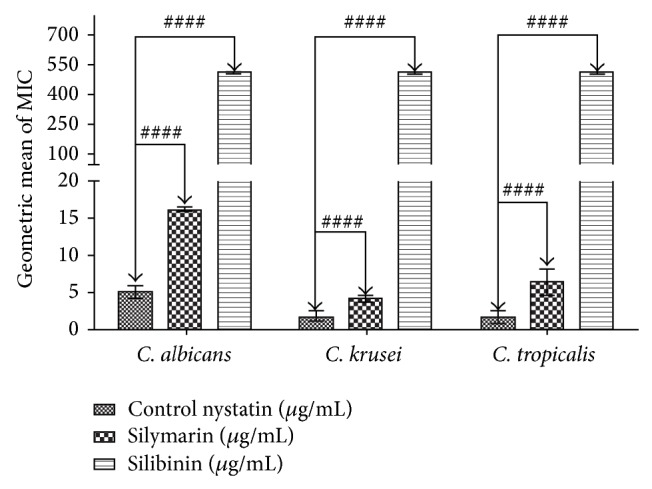
MIC (*μ*g/mL) of nystatin in the absence and presence of silymarin and silibinin at subinhibitory concentrations for* C. albicans, C. tropicalis*, and* C. krusei*.

**Figure 6:**
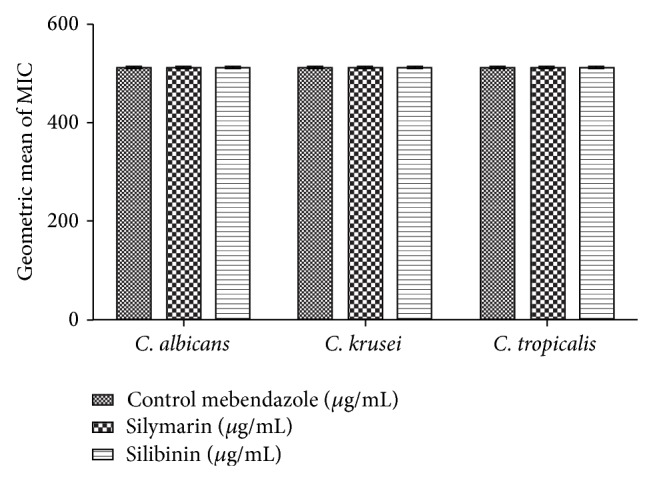
MIC (*μ*g/mL) of the mebendazole in the absence and presence of silymarin and silibinin at subinhibitory concentrations for* C. albicans, C. tropicalis*, and* C. krusei*.

The results demonstrating the modulatory effect against antifungal drugs were demonstrated in Figures 5 and 6. The antifungal modulatory activity of the products tested indicated an antagonistic effect against* C. albicans, C. tropicalis*, and* C. krusei*, when compared to nystatin and no significant effect in combination with mebendazole.

## 4. Discussion

Infections caused by pathogens such as* P. aeruginosa*,* S. aureus*,* E. coli*, and* C. albicans* have a high prevalence, where they are responsible for the increase in worldwide morbimortality of infections [27]. Factors involved in this increase vary from insufficient supply of antimicrobials, especially in poorer countries, to occurrence of antibiotic resistance. Thus, in the last decades, there has been an increase in the popular use of plants and their derivatives for infections caused by microorganisms [28].

Various studies on the evaluation of the antimicrobial activity of natural products have been conducted with the aim of broadening the spectrum of antimicrobial therapy. However, it is important to mention that the microdilution method, employed in the present investigation, currently represents the technique most accepted for this bioassay [29].

*Silybum marianum *(L.) Gaertn. (*Carduus marianus* L. Asteraceae) (milk thistle) has been used for more than 2000 years to treat liver and gallbladder disorders, including hepatitis, cirrhosis, and jaundice, and to protect the liver against poisoning from chemical and environmental toxins [12]. Silymarin is an active component of this plant, a standardized extract obtained from the seeds of* S. marianum* containing approximately 70 to 80% of the silymarin flavonolignans and approximately 20 to 30% is chemically undefined fraction, comprising mostly polymeric and oxidized polyphenolics compounds [30]. Silibinin is a major bioactive component of silymarin [13].

The incidence of studies investigating the biological activities of silymarin and silibinin has increased, given the variety of important pharmacological effects associated with these compounds, together with the fact that the use of silymarin/silibinin is considered safe, where there have been few reports of adverse effects [31, 32]. Recent* in vivo* and* in vitro* studies have demonstrated that silibinin has antioxidant, anti-inflammatory, antitumor, and antiarthritic properties [31]. Also, silibinin has shown antibacterial activity against the Gram-positive bacteria* Bacillus subtilis* and* Staphylococcus epidermidis *[33].

Findings have pointed to a synergistic drug-modifying effect when silymarin and silibinin were combined with antibiotics, especially aminoglycosides, against the different bacterial strains evaluated, where silibinin had an antagonistic effect when combined with imipenem and gentamicin against* P. aeruginosa*. Accordingly, phenolic compounds, for example, flavonoids and lignans, have demonstrated their therapeutic potential as antimicrobial agents, where they are considered responsible for this activity [34, 35]. The synergistic effect of flavonoids combined with commonly utilized antibiotics is well supported in the literature, emerging as an important complementary treatment modality in research [36].

It is believed, therefore, that phenolic compounds possess the capacity to form complexes with extracellular soluble proteins that bind to bacterial cell wall [37]. Studies have shown that many natural compounds alter the permeability of the cell membrane, favoring the penetration of antibiotics [38]. The interaction with bacterial enzymes can also be related to the synergistic mechanism of natural products with antibiotics [39], which can be obtained from an extract or from the combination of extracts, synthetic products, antibiotics, and other natural products [40, 41].

With respect to the antibacterial action of flavonoids, studies have demonstrated a significant inhibitory effect on DNA topoisomerase activity by the formation of complexes that alter enzyme binding [42]. In this perspective, the antibacterial activity of these compounds could also be related to the presence of hydroxyl phenolic groups that interfere with the bacterial synthetic processes by enzyme inhibition [43, 44].

Our results pointed to an antagonistic effect when silymarin and silibinin were combined with nystatin against the yeasts* C. albicans, C tropicalis*, and* C. krusei*. This result was probably due to the cell structure of the fungi, mainly the chitin cell wall of these microorganisms, which apparently affects the action of antifungal agents and drug-modifying activity of natural products. However, new studies are needed to determine how this occurs. Considering the growing use of antifungal agents in cancer treatment and infectious diseases in general, these agents have contributed to the increase in drug resistance, leading to the need to discover new and alternative treatment modalities [45]. Thus, plant species rich in active metabolites such as flavonoids merit attention [46].

## 5. Conclusions

This work indicates the possibility of the usage of silymarin and silibinin as a source of new drugs as adjuvants in the antibiotic therapy against multidrug resistant bacteria (MDR), being a promising choice against the concerning problem of the antibiotic resistance.

## Figures and Tables

**Table 1:** Origin of bacterial strains and their resistance to antibiotics.

Bacteria	Origin	Resistance profile
*Escherichia coli* 06	Surgical wound	Aztreonam, Amoxicillin, Ampicillin, Ampicillin, Amoxicillin, Cefadroxil, Cefaclor, Cephalothin, Ceftazidime, Ciprofloxacin, Chloramphenicol, Imipenem, Kanamycin, Sulphametrim, Tetracycline, and Tobramycin

*Pseudomonas aeruginosa* 03	Urine culture	Ceftazidime, Imipenem, Ciprofloxacin, Piperacillin-Tazobactam, Levofloxacin, Meropenem, and Ampicillin

*Staphylococcus aureus* 10	Surgical wound	Oxacillin, Gentamicin, Tobramycin, Ampicillin, Kanamycin, Neomycin, Paromomycin, Butirosin, Sisomicin, and Netilmicin

**Table 2:** Composition of* Silymarin *extract.

Compounds	*Silymarin *	LOD	LOQ
	mg/g	*μ*g/mL	*μ*g/mL
Gallic acid	2.16 ± 0.01^a^	0.009	0.029

Caffeic acid	5.09 ± 0.03^b^	0.032	0.105

Silybin A	12.75 ± 0.01^c^	0.011	0.034
Silybin B	15.93 ± 0.03^d^	0.027	0.89

Results are expressed as mean ± standard deviations (SD) of three determinations.

Averages followed by different letters differ by Tukey's test at *P* < 0.05.
